# Contextualizing critical thinking about health using digital technology in secondary schools in Kenya: a qualitative analysis

**DOI:** 10.1186/s40814-022-01183-0

**Published:** 2022-10-06

**Authors:** Faith Chesire, Marlyn Ochieng, Michael Mugisha, Ronald Ssenyonga, Matt Oxman, Allen Nsangi, Daniel Semakula, Laetitia Nyirazinyoye, Simon Lewin, Nelson K. Sewankambo, Margaret Kaseje, Andrew D. Oxman, Sarah Rosenbaum

**Affiliations:** 1 Institute of Health and Society, Faculty of Medicine, University of Oslo, Oslo, Norway; 2 Tropical Institute of Community Health and Development, Kisumu, Kenya; 3 School of Public Health, College of Medicine and Health Sciences, University of Rwanda, Kigali, Rwanda; 4 Department of Medicine, Makerere University, College of Health Sciences, Kampala, Uganda; 5 Centre for Epidemic Interventions Research, Norwegian Institute of Public Health, Oslo, Norway; 6 Faculty of Health Sciences, Oslo Metropolitan University, Oslo, Norway; 7 Department of Health Sciences Ålesund, Norwegian University of Science and Technology (NTNU), Ålesund, Norway; 8 Health Systems Research Unit, South African Medical Research Council, Cape Town, South Africa

**Keywords:** Critical thinking, Critical thinking about health, Critical health literacy, Health literacy, Kenya, Curriculum, Secondary school, Context analysis

## Abstract

**Background:**

Good health decisions depend on one’s ability to think critically about health claims and make informed health choices. Young people can learn these skills through school-based interventions, but learning resources need to be low-cost and built around lessons that can fit into existing curricula. As a first step to developing and evaluating digital learning resources that are feasible to use in Kenyan secondary schools, we conducted a context analysis to explore interest in critical thinking for health, map where critical thinking about health best fits in the curriculum, explore conditions for introducing new learning resources, and describe the information and communication technology (ICT) infrastructure available for teaching and learning.

**Methods:**

We employed a qualitative descriptive approach. We interviewed 15 key informants, carried out two focus group discussions, observed ICT conditions in five secondary schools, reviewed seven documents, and conducted an online catalog of ICT infrastructure in all schools (*n*=250) in Kisumu County. Participants included national curriculum developers, national ICT officers, teachers, and national examiners. We used a framework analysis approach to analyze data and report findings.

**Findings:**

Although critical thinking is a core competence in the curriculum, critical thinking about health is not currently taught in Kenyan secondary schools. Teachers, health officials, and curriculum developers recognized the importance of teaching critical thinking about health in secondary schools. Stakeholders agreed that Informed Health Choices learning resources could be embedded in nine subjects. The National Institute of Curriculum Development regulates resources for learning; the development of new resources requires collaboration and approval from this body. Most schools do not use ICT for teaching, and for those few that do, the use is limited. Implementation of Kenya’s ICT policy framework for schools faces several challenges which include inadequate ICT infrastructure, poor internet connectivity, and teachers’ lack of training and experience.

**Conclusion:**

Teaching critical thinking about health is possible within the current Kenyan lower secondary school curriculum, but the learning resources will need to be designed for inclusion in and across existing subjects. The National ICT Plan and Vision for 2030 provides an opportunity for scale-up and integration of technology in teaching and learning environments, which can enable future use of digital resources in schools. However, given the current ICT condition in schools in the country, digital learning resources should be designed to function with limited ICT infrastructure, unstable Internet access, and for use by teachers with low levels of experience using digital technology.

**Supplementary Information:**

The online version contains supplementary material available at 10.1186/s40814-022-01183-0.

## Key messages regarding feasibility

- What uncertainties existed regarding the feasibility?
  Is there a demand for teaching Kenyan secondary school students to think critically about health choices? Are any of the key concepts for making informed choices about health interventions being taught already? What are important factors to consider when implementing new learning resources in Kenyan schools? Can teachers and students access digital learning resources?
- What are the key feasibility findings?
  Education stakeholders in Kenya recognized the importance of secondary students learning to think critically about their health choices. Critical thinking and health both appear across subjects in the curriculum, but there are no direct links between the Informed Health Choices Key Concepts and the curriculum of any subjects in lower secondary school. The Kenya Institute of Curriculum Development (KICD) approves all learning content before it is made available to schools. Most schools do not use ICT for teaching, and for those few that do, use is very limited. Teachers have varying ICT experience.
- What are the implications of the feasibility findings for the design of the main study?
  Teaching critical thinking about health is possible within the current Kenyan lower secondary school curriculum. There is an interest in and need for learning resources, but they will need to be designed for inclusion in and across existing subjects. The KICD should be involved in development to provide guidance that can lead to resource suitability and approval. Digital resources will need to be designed in such a way that they do not require direct student access or constant Internet connectivity. They should be easy to use for teachers with limited ICT experience.

## Background

Individual and population health depend on choices people make, both personal health choices and policy decisions. In order for people to make good health decisions, they need to be able to obtain, process, and understand health information [1]. The world is overflowing with information, including claims or advice about treatments, that has the potential to affect health decisions positively or negatively [2]. For instance, the COVID-19 pandemic has been accompanied by an “infodemic:” “an over-abundance of information—some accurate and some not” [3]. Many people do not have capacity to assess the reliability of health claims to make good health decisions [2, 4, 5]. Kenya has included the right to health as a principle in the constitution of 2010 [6]. Further still, one of Kenya’s education goals is to instill in learners the value of well-being for self and others by providing appropriate knowledge and skills to make the right choices [7]. Currently, Kenya is replacing an outdated curriculum that prioritizes rote memorization and exam-based learning with a proposed competency-based curriculum aimed at developing skills and knowledge that can be applied to “real-life” situations. Critical thinking and problem-solving are among the competencies integrated into all levels of the curriculum with an intention of helping learners reason and make sound judgments [8]. Schools in the low- and middle-income countries have the potential to act as powerful behavior modifiers, not only by promoting specific, currently recommended health behaviors [9], but by teaching students how to appraise health claims they encounter in their daily lives, now and in the future.

People who are more engaged in their health are more likely to seek out and use the information to inform their health decisions. However, they need to have the skills necessary to assess the reliability of that information [9]. This is especially important in low-income countries, where people have few resources to waste on unreliable health claims. An international, multidisciplinary group has developed a framework for teaching people to think critically about health and choices [10]. This framework includes 49 Informed Health Choices (IHC) Key Concepts that are principles for evaluating the trustworthiness of treatment claims, comparisons (research evidence), and choices. They provide a starting point for developing learning resources that can help people assess health claims and make informed health choices and evaluation tools to measure people’s ability to do this [11].

A prior IHC project developed primary school resources to teach 12 IHC Key Concepts to children aged 10–12 years. A cluster-randomized trial involving 120 schools and 10,000 children in Uganda demonstrated a large effect on children’s ability to assess treatment claims [12], which was sustained for at least one year [13]. A process evaluation showed that teachers, children, parents, and education authorities found the resources important and relevant [14]. However, lack of time in school schedules for new content and the cost of printing the resources were barriers to scaling up their use.

These barriers could be overcome by developing digital resources that could fit into schools’ existing curricula. As a first step in developing and evaluating new, digital resources for teaching critical thinking about health to secondary school students, we conducted this context analysis to explore these and other factors that could impact the design, implementation, and successful use of such resources in Kenyan schools. The objectives were to (1) explore interest in teaching critical thinking for health, (2) map where critical thinking about health best fit in the curriculum, (3) explore conditions for introducing new learning resources in schools, and (4) describe what information and communication technology (ICT) facilities and software are likely to be accessible in Kenyan secondary schools for teaching and learning purposes, as well as national plans to improve what exists. Researchers in Rwanda and Uganda carried out similar context analyses [15, 16]

Our findings will inform the design, evaluation, and, if the resources are found effective, implementation of the IHC secondary school resources. Further, these findings will be relevant for policymakers, curriculum developers, and other education stakeholders who are developing or implementing digital learning resources and learning resources for critical thinking and health, particularly in low-income contexts.

## Methods

### Study design

We employed a qualitative descriptive approach in this study [17]. We collected data through document review, key informant interviews, focus group discussions, observations, and an online survey.

### Sampling

Our sampling combines data from both national and regional sources. Our Kenyan team is in Kisumu County; therefore, we recruited teachers and schools from this region. Kisumu has a mix of geographical locations (rural, urban) and school ownership (privately- owned, public, and faith-based) schools that is typical for the rest of Kenya.

For the document review, we purposively searched for national documents with relevant information pertaining to critical thinking, health, and ICT in secondary school education on the Kenyan government websites of education and ICT.

To capture the opinions, views, and experiences of a wide range of participants, we conducted 15 key informant interviews (KII) and two focused group discussions (FGD). For KIIs, we purposively sampled the 15 key informants relevant to our study objectives. These included national curriculum developers responsible for critical thinking and curriculum research (*n*=2), national ICT officer in charge of digital content (*n*=1), teachers from Kisumu County that use ICT for teaching or manage computer equipment (*n*=10), and national examiners (*n*=2) responsible for preparing national examinations for home science and biology.

For focus group discussions, we recruited teachers (*n*=12) and curriculum developers (*n*=9) as participants. The COVID-19 pandemic restrictions required schools to be closed during the study period. First, we purposively sampled two teachers within our neighborhood who then used snowballing and helped us identify nine teachers teaching subjects that cover health and critical thinking as part of their content and were available to participate. Those subjects, which we identified through document analysis of the curriculum, include mathematics, English, biology, chemistry, physics, agriculture, history, geography, home science, French, business studies, economics, Christian religious education, and social studies.

For the second focus group discussion, we contacted the Kenya Institute of Curriculum Development (KICD) and selected nine national curriculum experts in health-related subjects, critical thinking, and digital content. Their areas of expertise covered home science, mathematics, business studies, agriculture, biology, chemistry, and English, Christian religious education, and ICT.

We conducted an online survey and carried out observations of ICT conditions in secondary schools in Kisumu County. For the survey, we purposively included all the 250 secondary schools in Kisumu County, 229 publicly owned, and 22 privately owned, to assess the availability and state of ICT infrastructure used for teaching and learning. We sent emailed invitations to the school principals inviting them to participate in the survey. For observation of ICT infrastructure in schools, we purposively selected five schools that use ICT for teaching and learning. We then stratified the selected five schools based on ownership (private and public) and geographic location (rural and urban).

### Data collection procedures

#### Document analysis

For document review, we purposively downloaded and included the current and proposed curricula for lower secondary schools. The Curriculum Needs Assessment Report 2016 [18], national documents on ICT for education (Kenya Education Policy 2002 (7), and the National ICT Strategy for Education and Training 2006 [19]. Two independent researchers extracted statements pertinent to each study objective, summarized the findings, compared the data, and resolved any disagreement through discussion.

#### Key informant interviews and focus groups

For key informant interviews and focus group discussions, we used a semi-structured interview guide (Additional file 1). The interview guides included questions based on our study objectives. The objectives were broken down into themes that informed probes on critical thinking and health, assessment of critical thinking, ICT infrastructure, and the use of ICT in teaching. The interviews were conducted by three researchers: a moderator, a note taker, and an observer (FC, MOx, and CW). The moderator guided the discussion while the note taker took notes. The saturation point was based on the similarity of information for each theme. All interviews were conducted face-to-face in private rooms at the workplace of study participants who were assured of confidentiality and encouraged to express their opinions freely. We gave a basic description of what the study is about and sought written permission to record the discussions. The interviews lasted between an hour to one hour and a half.

#### ICT conditions in schools—online survey

We created an ICT survey checklist to assess the availability of ICT infrastructure for teaching and learning, as well as internet connectivity in schools (Additional file 2). We included all secondary schools (*n*=250) in Kisumu County. We started by seeking authorization from the county education office and obtained email contacts from school principals. The questionnaires were mailed to the principals who completed and returned them to the research team.

#### ICT conditions in schools—observation

We purposively selected five secondary schools within the county. These schools were selected based on four strata (rural-private (*n*=1), urban-private (*n*=1), rural-public (*n*=1), and urban-public (*n*=2)). We created a checklist to guide our observations (Additional file 3), based on parameters that our ICT-development team identified as important for understanding the use requirements for digital learning resources, drawing in part on relevant items from a set of educational ICT indicators [20]. We observed ICT conditions in schools and technology use in classrooms through non-participant observation during school visits.

### Data analysis

#### Document analysis

We reviewed the national policy documents and other Ministry of Education guidelines and reports (S1 Table) and evaluated texts in three steps: skimming, reading, and interpretation. This process entailed a first-pass document review, where meaningful and relevant passages of text were identified and then extracted [21]. We reviewed the current curriculum and proposed a basic curriculum framework for lower secondary schools in Kenya. We examined the course outlines and extracted subjects, topics, and objectives that indicated critical thinking, health, or critical thinking about health. We also examined the core competencies, objectives for health-related subjects and proposed assessment approaches for the proposed curriculum framework. We reviewed the recommendations of the curriculum needs and Kenya education reports and extracted recommendations on the need for critical thinking, health, and digital literacy. We reviewed policy documents for ICT in education, as well as strategic and implementation plans for ICT in schools, and extracted the policy goals and implementation status of ICT infrastructure in Kenyan secondary schools.

The amount of data we collected was guided by considerations of the variation in issues emerging from the data collection and the extent to which we were able to explain these variations, our time and resource constraints, coupled with the need to avoid large volumes of data that cannot be easily managed or analyzed as highlighted in the literature [22, 23]

#### Data analysis from interviews, focus groups, ITC observation and online survey

We collated and analyzed data from key informant interviews, focus group discussions, observation, and document review using a framework analysis approach for applied research [24]. This approach borrows from phenomenology. Various verification strategies, such as concurrent data collection and analysis, constant comparative analysis, and iterative analysis, serve to locate the findings within the framework of the existing body of knowledge [24].

We transcribed the audio data verbatim. We read through the transcripts to familiarize ourselves with the data sets. Two researchers independently read one sampled transcript from the datasets and coded relevant statements according to the study objectives. Thereafter, the researchers compared the identified codes for the study objectives and agreed on a standard coding framework for subsequent transcripts as shown in Table 1. The researchers charted the codes for each objective and compared and agreed on the summaries. We analyzed and interpreted the results in relation to the objectives. We analyzed the online survey data on ICT infrastructure status in schools using Microsoft Excel to calculate frequencies and proportions.

**Table 1:** Codes used to analyze data from document analysis, key informant interviews, and focus group discussions

Objective	Themes	Codes
1) Explore interest in teaching critical thinking for health	Interest in learning resources for teaching critical thinking about health in secondary schools in Kenya	• Education goal on critical thinking • Curriculum assessment on critical thinking and health • Need for critical thinking about health • Comparison of IHC Key Concepts curriculum objectives on health
	Opportunities for teaching critical thinking about health	• Proposed curriculum • Resources to teach critical thinking about health • Gaps in teaching critical thinking
2) Map where critical thinking about health best fits in the curriculum	Curricular links to critical thinking about health	• Implicit link to IHC Key Concepts • Teaching critical thinking and health • Subjects to integrate IHC Key Concepts • Challenges in teaching critical thinking
	Current learning resources used to teach critical thinking about health	• Resources used in teaching critical thinking about health • Critical thinking assessment
3) Explore conditions for introducing new learning resources in schools,	Decision-making about learning resources in schools	• Who regulates the use of learning resources • Process of introducing new learning resources • Capacity needs for introducing IHC resources • IHC content
4) Describe what information and communication technology (ICT) facilities and software are likely to be accessible in Kenyan secondary schools for teaching and learning purposes, as well as national plans to improve what exists	Current ICT facilities available for teaching in secondary schools	• Policy plans to support ICT use for teaching in schools • Available ICT conditions in schools • Challenges of using ICT

We summarized the key findings and assessed our confidence in these using a version of the GRADE-CERQual approach [25] modified for primary qualitative studies [14]. GRADE-CERQual is a systematic and transparent method for assessing the confidence in evidence from reviews of qualitative research and involving applying four components: methodological limitations, data adequacy, coherence, and relevance [26]. Although CERQual has been designed for findings emerging from qualitative evidence syntheses, the components of the approach are suitable for assessing findings from a single study with multiple sources of qualitative data. We modified the components slightly, as follows: (1) Methodological limitations: the extent to which there are concerns about the sampling and collection of the data that contributed evidence to an individual finding; (2) coherence of the finding: an assessment of how clear and compelling the fit is between the data and the finding that brings together these data; (3) adequacy of the data contributing to a finding: an overall determination of the degree of richness and quantity of data supporting a finding; and (4) Relevance: the extent to which the body of evidence supporting a finding is applicable to the context (perspective or population, phenomenon of interest, setting) specified in the study question.

Two of the study investigators (FC and MOx) applied the modified GRADE-CERQual approach to each study finding and made a judgment about the overall confidence in the evidence supporting the finding. We judged confidence as being high, moderate, low, or very low. All findings started as high confidence and were graded down if there were important concerns regarding any of the components described above [27].

## Results

We interviewed 15 key informants including policy makers (*n*=5) and teachers (*n*=10). We conducted two focus group discussions with curriculum developers (*n*=9) and teachers (*n*=12), respectively. We observed the ICT infrastructure for teaching and learning in five schools. We reviewed seven documents that included curricula and ICT for education. We sent the online survey questionnaires to all 250 secondary schools in Kisumu County and received responses from 175 schools. We judged all the key findings to have a CERQual assessment of “high confidence” [28]. These confidence assessments are reported in parentheses, following the corresponding finding statement, and are described in more detail in (Additional file 4).

### Interest in learning resources for teaching critical thinking about health in secondary schools in Kenya

Kenya has a national goal to promote health through education (high confidence). One of the objectives of the Kenyan national goal for education is to integrate health into education. The policy indicates a need to empower learners with skills and knowledge in health, and states that “Education should inculcate in the youth the value of good health in order to avoid indulging in activities that will lead to physical or mental ill health.”

Teachers, health officials, and curriculum developers recognized the importance of teaching critical thinking about health in secondary schools in Kenya, noting that it will empower the learners with skills to make informed health choices (high confidence). Curriculum developers suggested that many students are missing the necessary skills.

There are so many influences on choice of diet. Therefore, the teaching is very important to facilitate making informed choices. **Curriculum expert 1**.

Students require decision making skills and application of critical thinking, which are lacking among most of our learners. **Curriculum expert 2.**

Findings from the stakeholders are corroborated by reviews of the Kenya Curriculum Reforms Report [29] and the Curriculum Needs Assessment Report [18]. These reports identify the need to include critical thinking competencies and health subjects in the curriculum (high confidence). For instance, the Curriculum Reform Report recommends the need for a reformed curriculum that adopts a competency-based approach, with critical thinking and problem solving among the core competencies intended to enable the learners to apply learned skills in life situations. Additionally, the Curriculum Needs Assessment Report identifies health education as one of the missing pertinent issues affecting learners, recommending that it should be included in the proposed curriculum

Teachers pointed out that the IHC Key Concepts learning objectives appear to be in line with the goal of critical thinking competencies that are described by both the current and proposed competency-based curriculum (high confidence). For instance, the proposed curriculum framework describes critical thinking and problem solving as competencies that will enable learners: “to avoid subjectivity, to use logic and evidence in arriving at conclusions; facilitate learners to explore new ways of doing things and gain autonomy; take account of multiple perspectives when addressing issues; be open-minded to, listen and appreciate information and opinions that may sometimes conflict with their prior beliefs” [30]. However, teachers and curriculum experts indicated that it does not appear that these skills are being taught, as teaching is examination-focused, and examinations tend to assess rote memorization.

Furthermore, teachers and curriculum developers noted that the IHC learning resources could contribute to the achievement of the Kenya national education goals for critical thinking and health.

### Curriculum links to critical thinking about health

We explored how critical thinking about health is represented in the curriculum. Our review of the current curriculum and proposed curriculum framework found no direct link between IHC Key Concepts and any of the subjects in lower secondary school (high confidence). Our discussions with curriculum experts confirmed this finding. However, we found that critical thinking and health both appear across subjects in the curriculum (high confidence), with the intention of equipping learners with skills and knowledge that can support their health. Additionally, subjects like home science and mathematics have broad objectives that may implicitly link to the IHC learning objectives.

Interpret and use advertisements wisely; Identify sources of consumer information and Acquire awareness on Consumer Education and be able to utilize it wisely. **home science course outline.**

We reviewed Kenya’s current and proposed reformed curricula and found that both include health topics that provide opportunities for teaching IHC Key Concepts. Four (English, home science, mathematics, and business studies) of the total 14 subjects in the current curriculum host health topics (high confidence) (see Table 2). Home science and business studies include standalone health topics, while English and mathematics use health examples to illustrate other concepts in the curriculum.

**Table 2:** Health-related topics for lower secondary school

Subject	Level	Topics
*English*	Form 1	Reading (health passages): Recall, comprehension, application, and analysis; extensive reading on literary materials on contemporary issues
*Mathematics*	Form 1	Fractions
		Rates ratios, percentages, and proportions
	Form 2	Statistics (interpretation of data)
*Home science*	Form 1	Safety in the home and first aid; medicine use and abuse
		Personal hygiene (choice, use, and misuse of cosmetics)
	Form 2	Consumer education (importance of consumer education) Environmental hygiene (common communicable diseases) Advertisements; consumer awareness (effects of advertisements on consumers; sources of consumer information)
*Business studies*	Form 2	Government (need for and methods of consumer protection)

In the reformed curriculum, health is proposed taught as a standalone subject (health education) in addition to being included as a topic in three other subjects (home science, business studies, and physical education). Teachers and curriculum developers confirmed these findings while at the same time suggesting four additional subjects (biology, chemistry, Christian religious education, and geography) in which IHC Key Concepts could potentially be taught.

Health is a cross-cutting topic in many subjects. For example, in mathematics, the exercises cover health related aspects such as disease prevalence proportions. **Mathematics teacher.**

Health is a cross-cutting topic in subjects which may offer opportunities for linkage of IHC concepts. **Curriculum expert 4.**

Critical thinking is currently taught in Kenya using different approaches (high confidence). Teachers and curriculum developers indicated using methods such as exposing learners to open-ended questions, use of photos, songs, videos, and public speaking. However, our document review and interviews with teachers and curriculum developers suggest that critical thinking is only taught to a limited extent (high confidence). Some of the reasons for this include inadequate pre-service training, lack of guidance on how critical thinking should be taught, inadequate resources, a crammed school schedule, and fear that students will challenge teachers. The review of the current curriculum and findings from interviews showed that there were no available resources for teaching critical thinking about health (high confidence). The lack of these resources was cited as one of the reasons for the limited teaching of critical thinking about health.

It comes up as a debate. However, it is not sufficient. It is not emphasized, but it will be in the competency-based curriculum. **Mathematics teacher.**

The delivery approach is teacher-centered. Hence the learner expects the teacher to bring in the solutions. The content is also rigid. **Curriculum expert 3.**

According to the national examiners we interviewed, critical thinking assessment is not implemented in the current curriculum (high confidence). They reported that the Ministry of Education is phasing out the current curriculum—which they described as rigid, exam-oriented, and leading to rote learning—and will replace it with a reformed, competency-based curriculum to be rolled out from 2024.

The current examination has focused more on how much content students are able to give back. The proposed curriculum will test high order skills. **National home science examiner.**

They also reported that testing will shift to include critical thinking skills. The reformed curriculum describes how the formative assessment of competencies will be conducted as described in the quote below:

Competencies shall be assessed over a period of time using projects, journaling, profiles and portfolios. The teacher shall document the learner’s achievement that shall show the progress towards the achievement of the learning outcomes identified in the subjects using a rating scale. The teacher and other observers shall be trained on how to create criteria for the assessment of competencies. The assessment shall involve teachers, parents and other stakeholders who shall look for opportunities where the learners can apply the competency in all areas of their life. **Kenya curriculum framework.**

### Conditions for introducing new learning resources

The Kenya Institute of Curriculum Development (KICD) regulates learning resources that are used in schools by approving textbooks and digital learning content (high confidence). The approved resources are listed in what is referred to as the ‘Orange Book.’ Schools are not allowed to purchase textbooks outside the approved list. Subject teachers choose the resources needed from the KICD’s approved list.

KICD come up with suggested resources at the development stage. However, the teachers decide resources to use from the Orange Book. **Curriculum expert 5.**

The current school timetable presents a challenge for introducing new content in the curriculum (high confidence), as it is full, with no space for additional content during regular school hours. According to the Basic Education Act, official school hours for secondary school are from 8:00 am to 3:30 pm (7.5 h), per day; Monday to Friday, with an additional 1.25 h for games and clubs (from 3:30 pm to 4:45 pm) [31].

Teachers and curriculum developers that we interviewed suggested that new content, such as the IHC Key Concepts, could potentially be taught in school health clubs and games, although they felt it would be better to teach them during regular school hours. Further, they noted that the proposed curriculum may provide a better opportunity than the current curriculum for introducing the IHC Key Concepts, noting that it would likely be easier to incorporate new content as revisions before the new curriculum is implemented.

We explored how critical thinking about health should be taught with curriculum developers and teachers. They indicated that teachers should be provided with guidance, because they have little or no experience teaching the IHC Key Concepts (high confidence). They also suggested using current and relatable health claims that learners can understand in the learning resources, such as COVID-19 claims and context-specific reproductive health claims.

### Availability and use of ICT for teaching and learning

There are policies and guidelines to promote ICT in education (high confidence). Kenya has a progressive national ICT policy which aims to “encourage the use of ICT in schools, colleges, universities, and other educational institutions in the country to improve the quality of teaching and learning” [19]. However, according to teachers and digital content developers, the ICT policy plan to set up computer laboratories in all public secondary schools in the country has not been fully operationalized. They did note that digital literacy is incorporated across all subjects in the proposed competence-based curriculum [19] and presents an opportunity for use of digital learning resources.

**Fig. 1:**
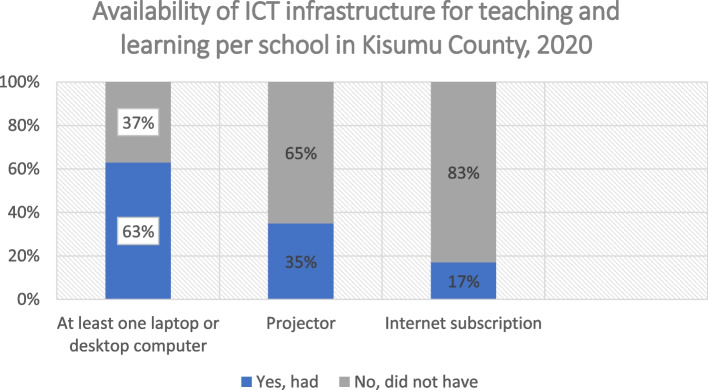
Availability of ICT infrastructure for teaching and learning in Kisumu County secondary schools, 2020. (Answers: per school, *N*= 175)

Most schools have inadequate ICT infrastructure for teaching and learning (high confidence). According to our survey, 63% of secondary schools had at least one laptop or desktop computer, 35% of the schools owned a projector, and only 17% had an internet subscription (see Fig. 1). Teachers we interviewed reported that students have limited access to computers. Teachers also mentioned that few students owned phones and students are not permitted to bring phones to school. In schools we visited, we observed some students using computers in groups, and some teachers using projectors to display digital learning materials to the class.

To cope with inconsistent Internet connectivity in schools, teachers described using their personal smartphone or computer to search for and download digital material. Then, they would either print the material or display it offline during class, for example using a projector, if available.

Sometimes we rely on our phone internet to complement the teaching resources, we use e-material downloaded from YouTube to explain concepts when teaching. For instance, in History topics such as World Wars, I download YouTube videos from my phone to complement theory. **History teacher that uses ICT**

Teachers that we interviewed said that reasons for low Internet subscriptions in schools included high subscription costs, power outages, and poor network connections. Additional challenges that were identified by teachers and are also mentioned in the National Council of Science and Technology report (2010) included teachers’ lack of ICT skills, lack of digital content, and fear of internet misuse, such as accessing pornography or cyberbullying (NCST, 2010).

We found that a variety of Internet browsers are in use, including Google Chrome, Microsoft Internet Explorer, Mozilla Firefox, and Opera Mini. Based on school visits, we did not identify any standard software applications used across schools.

## Discussion

This study explored factors that could inform and impact the development, implementation, and eventual use of digital learning resources for secondary schools in Kenya, focusing specifically on learning resources for teaching critical thinking about health. We found out that critical thinking is among the core competencies for both current and proposed lower secondary school curricula, but there is no explicit mention of competencies that map directly onto the IHC Key Concepts. Despite this, stakeholders (curriculum developers, policy makers, and teachers) express a clear interest in IHC learning resources, due to the perceived importance of the subject matter. Health and critical thinking are already taught across nine subjects and may provide an opportunity for IHC integration. However, many schools have limited ICT infrastructure for teaching and learning.

Two key findings support the conclusion that there is interest in learning resources for teaching critical thinking about health. Firstly, critical thinking is among the core competencies for both current and proposed reformed curricula for lower secondary schools, though to a lesser extent in the current curriculum. Secondly, key stakeholders identified critical thinking for health as a necessary competence for Kenyan young people. These findings are also shown in similar context analyses conducted in Uganda, Rwanda, and Norway ) [15, 16, 32].

Although the curricula do not explicitly mention IHC Key Concepts, we identified several four subjects where critical thinking about health could potentially be taught. This is also consistent with findings of context analyses in Uganda, Rwanda, and Norway where researchers identified several relevant subjects, but that varied from curriculum to curriculum [16] under review [15, 33]: Biology and health sciences, Chemistry, Mathematics, English, Home sciences, Physical education, and Food and health. Another example is from the Key Stage 3 National Science Curriculum for schools in England (for children between ages 11 and 14) that covers conceptual understanding, scientific inquiry, and the uses and implications of science. Researchers analyzing this curriculum found that some IHC Key Concepts were relevant for those themes and could help learners to understand what working scientifically entails [34] argues that continuous self-evaluation and self-improvement in primary education may have the effect of opening schools up to the possibility of introducing a program like IHC. Conversely, it may also temporarily shut the door to new programs like IHC if those programs do not fall within the area identified for self-improvement by the school.

To the extent that critical thinking is being taught, we found that teachers use various tools and strategies, including open-ended questions, multimedia, songs, debate, and public speaking. This is consistent with a systematic review that found that teachers use numerous instructional methods to promote thought and active learning in the classroom [35]. Our finding that critical thinking is only being taught to a limited extent is consistent with a critical analysis of Kenya’s approach to teaching critical thinking [36]. The authors of this analysis found that while the curriculum mentions developing critical thinking as one of its objectives, the education system prioritizes competitive examinations and rote memorization over critical thinking and other vital twenty-first-century skills. Studies have also found that teachers in Kenya have challenges teaching critical thinking due to their training [36, 37] and lack of suitable teaching resources. Our finding that critical thinking skills are not assessed is consistent with evidence elsewhere that critical thinking assessment has been neglected even more than critical thinking instruction [38]. The introduction of learning resources designed to help educators teach critical thinking about health, accompanied by evaluation tools that assess students’ acquisition of these critical thinking skills, would help move classroom practices and student outcomes in the desired direction.

There is a strict bureaucratic process for the approval of learning resources in Kenya. Teachers, in consultation with school heads, can decide which of the approved resources to use. This differs from some other countries, such as Norway, where teachers have more discretion about what and how they teach, not limited to approved learning resources [32]. This means that groups developing new learning resources with the intention of making them suitable for implementation across Kenyan schools should work in collaboration with the curriculum development office for guidance and input that can ensure approval.

In many schools, there is currently very limited ICT infrastructure for teaching and learning. These findings are consistent with Uganda context and other similar studies in developing countries on ICT challenges, which include limited electrical and internet infrastructure in rural areas, limited availability of technically skilled support staff, and under-qualified teaching staff [16, 39]. In contrast, the Rwandan showed that the government has provided computers, connectivity, and other ICT devices to more than 50% of schools for supporting teaching and learning. According to the REB ICT for the education department, over 50% of secondary schools in Rwanda have at least two smart classrooms and laptops for teachers in each department [15]. We also found that many teachers have inadequate ICT skills. Other studies have shown that ICT training, collaboration among teachers, and perceived self-efficacy influence classroom ICT use [40]. Action research in Kenyan elementary schools suggested that the use of pre-recorded DVD-based content and the use of teachers to lead the design and development of appropriate materials had the potential to successfully introduce digital learning resources in ICT-constrained schools [41].

Implications for our development of learning resources are to create solutions that can be utilized in schools with fewer resources and poor ICT infrastructure (e.g., accessed by teachers on their own phones) are not dependent on constant internet connections, and are easy to use by teachers with limited experience with digital technology.

### Strengths and limitation

A strength of this study is the use of multiple data sources including document review, key informant interview, focus group discussion, observation, and a survey. This enabled us to triangulate findings and provided a rich description of the context from multiple perspectives. Having more than one researcher independently code and analyze data and applying the adjusted CERQual approach for assessing confidence in the findings reduced the risk of bias.

The use of a purposive selection of study participants could limit the generalizability of findings. Limiting the ICT context to Kisumu County could also limit the generalizability of ICT status to other regions in Kenya with different circumstances.

## Conclusion

Teachers and curriculum developers recognized the importance of critical thinking about health for learners. Yet, this competence is not expressly stated in the current curriculum and both teachers’ practices and availability of resources for teaching critical thinking about health are limited. Kenya’s current lower secondary school curriculum includes four subjects with a direct link and an additional five subjects with a high potential for embedding content on critical thinking about health. The curriculum and introduction of learning resources are controlled nationally in Kenya, underscoring the importance of collaboration with the national curriculum institute. The use of ICT for teaching and learning is limited; challenges include a lack of infrastructure and teachers’ lack of ICT skills. However, the government has plans to equip all public schools with ICT infrastructure and to utilize digital learning resources. This, together with the implementation of the new competence-based curriculum could lead to improved conditions for implementing digital resources for teaching critical thinking about health, and other digital learning resources. In the meantime, schools and teachers would be best served with flexible, easy-to-access and easy-to-use solutions that are configured for low-ICT-resource settings.

## Supplementary Information

**Additional file 1.** Interview guides.

**Additional file 2.** ICT survey checklist.

**Additional file 3.** ICT observation checklist.

**Additional file 4.** CERQUAL assessment of key findings for context analysis.

**Additional file 5.** COREQ checklist.

## Data Availability

The dataset supporting the conclusions of this article is available in the Norwegian Data Center repository at: http://nsddata.nsd.uib.no/webview/index.jsp?node=0&submode=ddi&study=http%3A%2F%2F129.177.90.161%3A80%2Fobj%2FfStudy%2FNSD2942&mode=documentation&top=yes
