# Supplementary material for: Contextualizing critical thinking about health using digital technology in secondary schools in Kenya: a qualitative analysis
Source: Pilot Feasibility Stud. 2022 Oct 6;8:227. doi: 10.1186/s40814-022-01183-0 (PMC9535840; doi:10.1186/s40814-022-01183-0)
Supplement: Supplementary file 1 — Additional file 1. Interview guides. [file 40814_2022_1183_MOESM1_ESM.docx]

**KII/ FGD INTERVIEW GUIDE: (curriculum developers and teachers)**

| IHC CHOICE – Context analysis interviews | | |
| --- | --- | --- |
| **Date** |  |  |
| **Name** |  |  |
| **Position** |  |  |
| **Interview no.:** |  |  |
| **Interviewer initials:** |  |  |
| **Audio recording?** |  |  |

**Topic 1*:***

**Teaching critical thinking, health, and critical thinking about health. Definition of critical thinking.**

*Prompts*

- What is critical thinking?
- Is there need/demand to teach it? Currently and in future?
- Why should it be taught?
- How is critical thinking taught today? (Skills/ its applicatication,)
- How is it offered? ( stand alone/ crosscutting)
- How is assessed today and in the future?
- How is health taught today?
- How is critical thinking about health being taught today? (Skills/ its applicatication)
- What need is there for teaching critical thinking about health?
- Where in the curriculum can it fit in?
- How much time could potentially be made available to teach critical thinking about health and how/where? (What could it replace?)
- What plans are there for developing the national curriculum with respect to critical thinking, health, and critical thinking about health?
- What are challenges to teaching students critical thinking about health?

**Topic 2:**

**Learning resources for teaching critical thinking, health and critical thinking about health**

*Prompts*

- What resources are currently used to teach these subjects?
- Who makes decisions about which learning resources to use and how?
- Where are learning resources typically found or accessed by teachers?
- Who are the decision makers?
- Is there guidance/criteria/standards for developing or approving new learning resources
