# Supplementary material for: Contextualizing critical thinking about health using digital technology in secondary schools in Kenya: a qualitative analysis
Source: Pilot Feasibility Stud. 2022 Oct 6;8:227. doi: 10.1186/s40814-022-01183-0 (PMC9535840; doi:10.1186/s40814-022-01183-0)
Supplement: Supplementary file 3 — Additional file 3. ICT observation checklist. [file 40814_2022_1183_MOESM3_ESM.docx]

**OBERSAVATION CHECKLIST (**seeing ICT in use, in addition to interviews)

| **Date** |  |
| --- | --- |
| **School Name** |  |
| **Level of school (National, County, Subcounty N/A)** |  |
| **School ownership(Private/ public)** |  |
| **School geographical location (Rural/Urban)** |  |
| **Observer Name:** |  |
| **Serial Number** |  |

1. **Description of what technologies you see in use (see previous page, 3A)**

| **Equipment** | **Number Available?** | **Devices mentioned (Year of make)** | **Your comment** | |
| --- | --- | --- | --- | --- |
|  |  |  | Old | New |
| Computers |  |  |  |  |
| Projectors |  |  |  |  |
| Smartphones |  |  |  |  |
| Tablets |  |  |  |  |
| Camera |  |  |  |  |
| Recorders |  |  |  |  |
| Electronic boards |  |  |  |  |
| Public Address System |  |  |  |  |
| Scanners |  |  |  |  |
| Television with video input devices (DVD, Flash drive) |  |  |  |  |
| Other, specify |  |  |  |  |
| No ICT devices |  |  |  |  |

1. **Who owns the devices(do students or teachers own the devices or do they rely on what is provided at school ?**

| Devise | Number available  Observe | **Who owns the devices** | Number of students (If applicable) | Number of teachers (While using the devices) | Do they use these devices individually and/or in groups (Individual, Group, Both) (Observe) |
| --- | --- | --- | --- | --- | --- |
| Computers |  |  |  |  |  |
| Projectors |  |  |  |  |  |
| Smartphones |  |  |  |  |  |
| Tablets |  |  |  |  |  |
| Camera |  |  |  |  |  |
| Recorders |  |  |  |  |  |
| Electronic boards |  |  |  |  |  |
| Public Address System |  |  |  |  |  |
| Scanners |  |  |  |  |  |
| Television with video input devices (DVD, Flash drive) |  |  |  |  |  |
| Other, specify [TEXT] |  |  |  |  |  |

1. **What operating system(s) *(e.g. Windows, OS or Linux on desktop/laptop computers; iOS, Android, Chrome OS on tablets/smartphones)*? What version(s)? are currently used (Observe)**

| Devise | **Which operating system (OS(Mac), Windows, Linux, Android, Chrome Others specify**  **(Choose all that apply or specify**) | **What type/version of browsers *(e.g. Internet Explorer 9) does the school use on the student used devices?***  **Mozilla, Opera, Safari, Chrome, Internet explorer 9, others specify** | **Is there capacity to change or update the browsers**    **(1 Yes-change,**  **2 Yes- Update,**  **3 Yes- both,**  **0 No)** |
| --- | --- | --- | --- |
| Computers |  |  |  |
| Projectors |  |  |  |
| Smartphones/ phone |  |  |  |
| Tablets |  |  |  |
| Camera |  |  |  |
| Recorders |  |  |  |
| Electronic boards |  |  |  |
| Public Address System |  |  |  |
| Scanners |  |  |  |
| Television with video input devices (DVD, Flask drive) |  |  |  |
| Other, specify [TEXT] |  |  |  |

1. **Description of how these technologies are being used by teachers and by students**.

a). Describe the environment,

b). Describe the objects within the room( chairs, desks)

c). How is learning resources distributed (downloaded? Uploaded? Paper handouts)

d). Describe their interactions with each other and with the technology (teacher /students)

e). Describe their observable experiences including barriers and work-arounds, your thoughts and comments. (Use more paper to write or draw).

i. Barriers and workarounds

ii Comments / thoughts

f). Describe the sitting arrangements, (groups of stutent- computer and spacing estimates

g). Information security systems

h). Physical security of ICT /computer lab
