# Supplementary material for: Contextualizing critical thinking about health using digital technology in secondary schools in Kenya: a qualitative analysis
Source: Pilot Feasibility Stud. 2022 Oct 6;8:227. doi: 10.1186/s40814-022-01183-0 (PMC9535840; doi:10.1186/s40814-022-01183-0)
Supplement: Supplementary file 5 — Additional file 5. COREQ checklist. [file 40814_2022_1183_MOESM5_ESM.docx]

COREQ (COnsolidated criteria for REporting Qualitative research) Checklist

A checklist of items that should be included in reports of qualitative research. You must report the page number in your manuscript where you consider each of the items listed in this checklist. If you have not included this information, either revise your manuscript accordingly before submitting or note N/A.

| **Topic** | **Item No.** | **Guide Questions/Description** | **Reported on**  **Page No.** |
| --- | --- | --- | --- |
| **Domain 1: Research team**  **and reﬂexivity** | | | |
| *Personal characteristics* | | | |
| Interviewer/facilitator | 1 | Which author/s conducted the interview or focus group? | Page 9 |
| Credentials | 2 | What were the researcher’s credentials? E.g. PhD, MD | Page 9-10 |
| Occupation | 3 | What was their occupation at the time of the study? | page10 |
| Gender | 4 | Was the researcher male or female? | page10 |
| Experience and training | 5 | What experience or training did the researcher have? | page 9 |
| *Relationship with*  *participants* | | | |
| Relationship established | 6 | Was a relationship established prior to study commencement? | Page 10 |
| Participant knowledge of  the interviewer | 7 | What did the participants know about the researcher? e.g. personal  goals, reasons for doing the research |  |
|  |  |  | page 10 |
|  |  |  |  |
| Interviewer characteristics | 8 | What characteristics were reported about the inter viewer/facilitator?  e.g. Bias, assumptions, reasons and interests in the research topic |  |
|  |  |  | Page 10 |
|  |  |  |  |
| **Domain 2: Study design** | | | |
| *Theoretical framework* | | | |
| Methodological orientation and Theory | 9 | What methodological orientation was stated to underpin the study? e.g. grounded theory, discourse analysis, ethnography, phenomenology,  content analysis |  |
|  |  |  | Page 11 |
|  |  |  |  |
| *Participant selection* | | | |
| Sampling | 10 | How were participants selected? e.g. purposive, convenience,  consecutive, snowball |  |
|  |  |  | Page 7-8 |
|  |  |  |  |
| Method of approach | 11 | How were participants approached? e.g. face-to-face, telephone, mail,  email |  |
|  |  |  | Page 6,10 |
|  |  |  |  |
| Sample size | 12 | How many participants were in the study? | Page 13 |
| Non-participation | 13 | How many people refused to participate or dropped out? Reasons? | N/A |
| *Setting* | | | |
| Setting of data collection | 14 | Where was the data collected? e.g. home, clinic, workplace | Page 9-10 |
| Presence of non-  participants | 15 | Was anyone else present besides the participants and researchers? |  |
|  |  |  | Page no |
|  |  |  |  |
| Description of sample | 16 | What are the important characteristics of the sample? e.g. demographic  data, date |  |
|  |  |  | Page 7 |
|  |  |  |  |
| *Data collection* | | | |
| Interview guide | 17 | Were questions, prompts, guides provided by the authors? Was it pilot  tested? | Page 9 |
|  |  |  |  |
| Repeat interviews | 18 | Were repeat inter views carried out? If yes, how many? | N/A |
| Audio/visual recording | 19 | Did the research use audio or visual recording to collect the data? | Page 9 |
| Field notes | 20 | Were field notes made during and/or after the interview or focus group? | Page 9 |
| Duration | 21 | What was the duration of the inter views or focus group? | Page 10 |
| Data saturation | 22 | Was data saturation discussed? | Page 9 |
| Transcripts returned | 23 | Were transcripts returned to participants for comment and/or | N/A |
